# Effects of Dietary Protease Levels on Growth Performance, Feeding Regulation, Glucose and Lipid Metabolism, and Endogenous Protease Secretion in Chinese Perch (*Siniperca chuatsi*)

**DOI:** 10.3390/ani15192809

**Published:** 2025-09-26

**Authors:** Shizhen Liu, Yi Yi, Qingda Tian, Jianmei Su, Liwei Liu

**Affiliations:** 1 Hubei Key Laboratory of Regional Development and Environmental Response, Faculty of Resources and Environmental Science, Hubei University, Wuhan 430062, China; 202421108012135@stu.hubu.edu.cn; 2 College of Fisheries, Chinese Perch Research Center, Engineering Research Center of Green Development for Conventional Aquatic Biological Industry in the Yangtze River Economic Belt, Ministry of Education, Huazhong Agricultural University, No.1, Shizishan Street, Hongshan District, Wuhan 430070, China; HZNYDXYY@163.com (Y.Y.); tianqingda@outlook.com (Q.T.)

**Keywords:** Chinese perch (*Siniperca chuatsi*), deaminase genes, feeding behavior, feed utilization, protease, glycolipid metabolism

## Abstract

300 healthy Chinese perch (*Siniperca chuatsi*) (34.35 ± 0.47 g) were randomly divided into five groups (P1–P5) fed diets supplemented with 0, 0.2, 0.4, 0.8, and 1.6 g/kg protease for 8 weeks. Compared to P1, protease supplementation significantly up-regulated endogenous pepsinogen genes (*pga1* and *pgc*) and down-regulated the muscle deamination gene *ampd*. In comparison to P1, the expression level of the hepatic gene *ast* increased in P2, P3, and P5, while *gdh* elevated in P2 and P3 (*p* < 0.05). Compared to P1, the expression of feeding-related gene *npy* decreased while *pomc* increased in P2; *agrp* increased in P3; and *pomc* and *cart* decreased in P5, resulting in significant increases in feed intake in P2, P3, and P5 (*p* < 0.05). Glycolytic genes (*gk* and *pk*) and lipid metabolism gene *pparα* were up-regulated in P2, P3 and P5, while *hsl* increased in P3 but decreased in P5 (*p* < 0.05). P5 exhibited significantly improved weight gain rate, specific growth rate, protein efficiency ratio, and protein retention rate, alongside reduced feed conversion ratio compared with P1. Therefore, dietary 1.6 g/kg protease significantly enhances growth, improves feed efficiency, stimulates pepsinogen secretion, and modulates deamination, glycolytic, and lipid metabolism genes in *Siniperca chuatsi*.

## Simple Summary

Chinese perch (*Siniperca chuatsi*) aquaculture is constrained by poor artificial feed acceptance and high protein demands. While exogenous proteases enhance nutrient utilization, their optimal dosage and mechanism in this species were unknown. This study systematically investigated graded neutral protease levels (0–1.6 g/kg) over 8 weeks on growth, feed utilization, and key gene expression. Our results illustrated that 1.6 g/kg protease significantly improved weight gain, specific growth rate, feed intake, and protein retention while reducing the feed conversion ratio of Chinese perch (*p* < 0.05). Crucially, this dose stimulated appetite by suppressing anorexigenic genes (*pomc* and *cart*), enhanced glycolytic (*gk* and *pk*) and lipid metabolic gene (*pparα*) expression leading to more efficient energy utilization, and maximally up-regulated endogenous pepsinogen genes (*pga1* and *pgc*), indicating improved protein digestion in feed. This study identifies that the addition of 1.6 g/kg protease to feed can effectively enhance the growth performance of Chinese perch by regulating the expression of appetite-regulating genes, glucose and lipid metabolism-related genes, pepsinogen genes, and deaminase genes. It provides an important theoretical basis for the development of artificial feed for Chinese perch and the improvement of aquaculture efficiency.

## 1. Introduction

With the development of the aquaculture industry, the reduction in marine resources and the restriction of fishing ban regulations have resulted in an insufficient supply of high-quality fish meal to meet market demands [1]. Plant protein sources are considered to be an ideal substitute for fishmeal in fish feeds due to their abundant resources, stable supply, low price, high protein content, environmental friendliness, and no risk of animal-derived pathogens [2,3]. However, plant-based protein feeds contain various antinutritional factors (ANFs), including phytates, non-starch polysaccharides, and protease inhibitors. These ANFs not only reduce feed palatability and fish intake, but also stress the fish gastrointestinal tract and impair protein digestion and absorption [4,5]. Consequently, they decrease fish immunity and growth performance. The development of novel feed additives is important for the sustainable and green development of aquaculture by enhancing the growth performance, overall health, and nutritional quality of aquaculture animals to meet the growing global demand for aquatic products [6,7].

Proteases are a class of biocatalysts that catalyze the hydrolysis of complex proteins into small molecule peptides and free amino acids [8,9] and are also widely used as multifunctional feed additives in aquaculture feeds. Currently, the main proteases commonly used in aquaculture feeds are neutral and acidic proteases [10]. On the one hand, protease supplements can effectively eliminate antinutritional factors in feeds, hydrolyze complex proteins through enzymatic reactions, and improve the consumption of dietary amino acids [11,12]. On the other hand, amino acids or peptides decomposed by proteases can influence deamination and glycolipid metabolism in fish, reduce the emission of nitrogenous compounds, and provide energy for fish growth [13,14]. Studies have shown that the addition of proteases to Nile tilapia (*Oreochromis niloticus*) and Common carp (*Cyprinus carpio*) feeds can enhance fish growth performance by increasing the digestibility and improving the palatability of plant protein feeds [15,16]. For instance, the addition of 1250 U/kg and 1100 U/kg of protease significantly improved the growth performance of juvenile African catfish (*Clarias gariepinus* (B.)), respectively [17]. Furthermore, the addition of protease to fish feed can enhance fish feeding, improve their feed utilization and digestibility, and enhance fish immunity, thus promoting growth performance and disease resistance [15,18,19,20,21].

Endogenous digestive enzyme activity in fish is an important indicator reflecting the digestive physiology of fish, and pepsin activity in carnivorous fish is usually higher than that in phytophagous fish to accommodate the digestive demands of high-protein diets [22]. The endogenous enzymes in the digestive tract primarily include alkaline proteases, lipases, acid proteases, and amylases, and their secretion in fish is often related to feed composition, fish species, growth stage, and environmental factors [23,24]. Studies have shown that the supplementation of proteases in feeds can stimulate the secretion of endogenous proteases in fish, further enhancing their digestion and absorption of nutrients [20,25,26,27]. The addition of 0.15 g/kg and 0.3 g/kg protease to the feed significantly increased the protease activity in the intestinal and hepatopancreatic tissues of juvenile gibel carp (*Carassius auratus gibelio*) [11]. Addition of a multi-enzyme complex (amylase (800 U/kg), acid protease (300 U/kg), and papain (3800 U/kg)) to the feed significantly enhanced the activities of pepsin and gastric amylase in snakehead (*Channa argus*) [28]. Adding 1.5–2.0 g/kg of enzyme complexes (neutral protease, acid protease, and glucoamylase, etc.) to the diet of Japanese seabass (*Lateolabrax japonicus*) promoted liver and intestinal endogenous enzyme (amylase activity, lipase activity, and trypsin) secretion [29]. Therefore, dietary protease supplementation can compensate for the deficiency of endogenous enzymes, particularly in young animals, aiding in the breakdown of indigestible macromolecules of protein [25].

The addition of proteases to feed plays a crucial role across various fish species and their developmental stages. Compared to omnivorous and herbivorous fish, carnivorous fish exhibit a higher demand for dietary protein content, thus potentially requiring greater supplementation of proteases in their feed to aid digestion and absorption [9,30]. A large amount of literature suggests that the addition of different concentrations of proteases can promote fish growth and metabolism. For instance, supplementing 1 g/kg of protease to a 50% high-protein distiller’s grain-based fish feed for European seabass (*Dicentrarchus labrax*) significantly improved body weight, specific growth rate, and food intake [31]. Similarly, adding 0.25 g/kg protease to Nile tilapia (*O. niloticus*) feed and 0.25 g/kg complex enzyme (phytase, protease, α-amylase, lipase, cellulase, etc.) to the feed of great sturgeon (*Huso huso*) significantly enhanced their growth performance [32,33], respectively. However, the addition of excessive proteases can also negatively affect fish growth. It was found that supplementing 0.2–0.4 g/kg neutral protease significantly increased the apparent digestibility coefficient, promoted the intestinal villi development, and improved intestinal structure in Nile tilapia (*O. niloticus*), whereas adding 0.8 g/kg protease resulted in decreased the apparent digestibility coefficient and damage to the intestinal villi [26]. Therefore, the optimal amount of protease added to feed in different fish species needs to be further investigated.

Chinese perch (*Siniperca chuatsi*) is a carnivorous fish that feeds on live fish and has a high protein requirement in its diet, which can be acclimated to artificial feeds [34,35]. Currently, the development of formulated feed for Chinese perch faces numerous challenges, such as low acceptance of artificial feed, high and difficult-to-balance protein requirements, and stringent demands for feed palatability. These factors severely limit the feeding, digestion, absorption, and growth of Chinese perch, while the amount of protease added to Chinese perch artificial feeds remains unknown. Therefore, this study focuses on Chinese perch as the research subject, supplementing various concentrations (0–1.6 g/kg) of neutral protease into its diet for 8-week feeding trial to investigate the effects and mechanisms of dietary protease on the growth, feeding, glucose and lipid metabolism, secretion of endogenous proteases, and protein utilization of Chinese perch. The aim of this study was to evaluate the effects of different protease concentrations on the feed, promote the growth performance of Chinese perch, and provide a theoretical basis for the application of proteases in aquaculture.

## 2. Materials and Methods

### 2.1. Preparation of Experimental Diets

In this study, five groups of isonitrogenous and isoenergetic Chinese perch (*S. chuatsi*) feeds were formulated using fish meal, corn protein powder, cornstarch, and amino acid premix as protein sources and fish oil as the fat source. The specific experimental feed formulations and nutritional compositions are shown in Table 1. The protease added in feed was a neutral protease (EC3.4.23.18), which was produced by *Humicola* sp. L8 fermentation (Wuhan SunHY Biology Co., Ltd., Wuhan, China) [26]. The enzyme activity was 1.38 × 10^7^ U/kg. The protease concentrations added to the feeds in this study were 0.0 g/kg (0.0 U/kg), 0.2 g/kg (2766 U/kg), 0.4 g/kg (5532 U/kg), 0.8 g/kg (11,064 U/kg), and 1.6 g/kg (22,128 U/kg), which were designated as P1, P2, P3, P4, and P5, respectively. The concentration of protease supplemented in the feed referenced effective levels reported in published studies on gibel carp (*C. gibelio*) [11,25], Japanese seabass (*L. japonicus*) [29], European seabass (*D. labrax*) [31], Nile tilapia (*O. niloticus*) [19,26], and common carp (*C. carpio*) [16], with the added concentration around 0–1.5 g/kg. In this study, P1 was set as the control group. The feed was thoroughly mixed and then processed into pellet feed (5 mm) using a pellet mill (Zhengchang Feed Machinery Co., Ltd., Liyang, China). The feed was subsequently grouped and stored in a −20 °C freezer.

### 2.2. Feeding Management of Experimental Chinese Perch

Chinese perch were purchased from the Xianning Haihui Aquaculture Cooperative (Xianning, China) and were acclimatized for 14 days at the Aquaculture Research Facility of Huazhong Agricultural University (Wuhan, China). All procedures were conducted in accordance with the animal protocol approved by the Ethics Committee of the Institute of Laboratory Animal Centre, Huazhong Agricultural University (protocol code: HZAUFI-2023-001, 10 May 2023). During the acclimation period, the fish were acclimated to feed on a formulated diet (P1) [36,37]. Three hundred healthy Chinese perch with uniform size (34.35 ± 0.47 g), robust physique, no injuries, and stable feeding status were selected. The fish were anesthetized using final concentration of 200 mg/L MS-222 (Sinopharm Chemical Reagent Co., Ltd., Beijing, China) [37,38], then weighed, and their initial body weight was recorded. Subsequently, randomly distribute the experimental fish into 15 disinfected culture tanks (1.6 m in diameter, 0.9 m in height), with 3 replicated tanks per group (20 fish per tank) and 5 groups in total, for an 8-week culture experiment. Feed the fish daily at 9:00 AM, with the amount of feed being approximately 3% of the fish’s body weight. After feeding, remove and weigh the residual feed to record the feed intake, and replace 1/3 to 1/2 of the water. During both the temporary rearing period and the farming period, it is necessary to regulate the aquaculture water quality to maintain dissolved oxygen at around 9 mg/L, water temperature between 19 and 24 °C, and pH between 7.8 and 8.1 [39,40]. Furthermore, the domestication conditions were consistent with normal farming conditions, except for the feeding ration.

### 2.3. Sample Collection

After conducting the 8-week Chinese perch farming experiment, prior to sampling, fish were fasted for 24 h. Subsequently, the sampled Chinese perch were anesthetized with 200 mg/L MS-222 (Sinopharm Chemical Reagent Co., Ltd., Beijing, China), then weighed, and recorded. Nine fish were taken from each group, and their body surfaces were rinsed with 0.9% physiological saline. Subsequently, the fish were dissected using sterile surgical scissors, with all procedures performed on ice. The brain, liver, stomach, and muscle tissues (dorsal muscle) were quickly removed [35] and placed into sterile centrifuge tubes. These samples were rapidly frozen in liquid nitrogen and subsequently stored at −80 °C in a freezer for further gene expression analysis [38,41].

### 2.4. Calculation of Growth Performance

Through the recorded feed intake, feed composition, initial body weight of Chinese perch, and final body weight obtained during sampling in the aquaculture experiment phase, the growth and feeding-related indices of the experimental Chinese perch during the aquaculture experiment phase were calculated: weight gain rate (WGR), specific growth rate (SGR), feeding rate (FR), feed conversion ratio (FCR), protein efficiency ratio (PER), and protein retention value (PRV). The calculation formulas for each index are as follows:

Weight gain rate (WGR, %) = (final weight − initial weight)/initial weight × 100%(1)

Specific growth rate (SGR, %) = (Ln final weight − Ln initial weight)/days × 100%(2)

Feed rate (FR, %) = feed intake/((final weight + initial weight)/2 × days) × 100%(3)

Feed conversion rate (FCR, %) = feed intake/weight gain × 100%(4)

Protein efficiency ratio (PER, %) = weight gain/protein intake × 100%(5)

Protein retention value (PRV, %) = protein gain /protein intake × 100%(6)

### 2.5. Analysis of Crude Protein

Nine fish were randomly taken from each group (three fish from each tank), and the crude protein content of the whole-body was determined using the Kjeldahl method [42,43].

### 2.6. Gene Expression Quantification

Nine fish were taken from each group (three fish from each tank), and total RNA was extracted from the brain, liver, stomach, and muscle tissues of Chinese perch using the RNAiso Plus kit (Takara, Dalian, China). RNA quality, integrity, and concentration were examined using 1.0% agarose gel electrophoresis and an ultra-micro NanoDrop 2000 spectrophotometer (Thermo Scientific, Waltham, MA, USA) [44]. For the qualified RNA samples (A_260_:A_280_ = 1.8–2.2), they were diluted to a concentration of 200–1500 ng/μL, followed by reverse transcription to synthesize cDNAs using the Evo M-MLV Reverse Transcription Premixed Kit (Accura Biotechnology, Changsha, China), and the synthesized cDNAs were stored in a refrigerator at −80 °C [45].

Primers for each gene (see Table 2 for primer sequences) were designed by Primer Premier 6.0 software and synthesized by Sangon Biotech Co., Ltd. (Shanghai, China). The cDNA was used as a template for qRT-PCR reaction using a Roche Light Cycler 480^®^ real-time PCR instrument (Roche, Indianapolis, IN, USA). Its reaction system (20 µL) contained the following components: 10 μL of 2 × ChamQ Universal SYBR qPCR Master Mix, 0.4 μL of upstream primer, 0.4 μL of downstream primer, 1 μL of cDNA, and 8.2 μL of diethylpyrocarbonate in water (Novozymes, Nanjing, China). The reaction program was set as follows: 95 °C, 30 s; 95 °C, 5 s; 40 cycles were performed; 60 °C for 30 s; and the lysis curve phase was from 65 to 95 °C (0.5 °C increments, 5 s/step) [40]. Three replicates of the qRT-PCR reaction were set for each gene, and the expression level of each gene was quantified by the 2^−ΔΔCT^ method [46]. Considering ribosomal protein L13a (*rpl13a*) exhibits a stable mRNA transcriptional level, the relative expression of each gene was detected by normalizing with the internal reference gene *rpl13a* [37,38].

### 2.7. Statistical Analysis

All data in this experiment were expressed as mean ± standard error (mean ± SE) and visualized using GraphPad Prism 9.0 software. First, samples deviating from the population mean were excluded using a one-sample *t*-test (α = 0.05) performed with IBM SPSS Statistics 27.0 software. Then, using one-way ANOVA and Duncan’s test analyzed the significance of differences between groups. The significance level was set at *p* < 0.05, and if the groups were labeled with different lowercase letters, this meant that the differences between groups were significant (*p* < 0.05). If the groups were labeled with the same lowercase letters, it meant that the differences between groups were not significant (*p* > 0.05).

## 3. Results

### 3.1. Growth Performance and Feed Utilization of Chinese Perch

**Figure 1:**
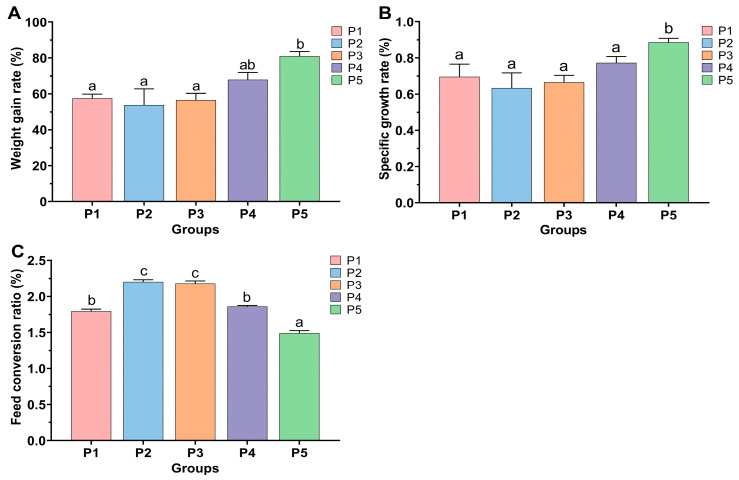
Effects of protease supplementation levels in feed on weight gain rate (**A**), specific growth rate (**B**), and feed conversion ratio (**C**) of Chinese perch. Different lowercase letters indicate significant differences among groups (*p* < 0.05), while identical lowercase letters indicate no significant difference (*p* > 0.05).

The effect of protease supplementation in feed on the growth performance of Chinese perch is shown in Figure 1. Compared to the weight gain rate (WGR, 57.54 ± 2.29%) and specific growth rate (SGR, 0.70 ± 0.07%) of the control P1 group, there were no significant differences in WGR and SGR among P2, P3, and P4 groups (*p* > 0.05). Only the P5 group showed a significant increase in WGR (80.99 ± 2.66%) and SGR (0.88 ± 0.02%) (*p* < 0.05) (Figure 1A,B). Compared to the feed conversion ratio (FCR, 1.80 ± 0.03%) of the control P1 group, the FCR of P2 and P3 groups (2.20 ± 0.03% and 2.18 ± 0.03%, respectively) significantly increased (*p* < 0.05). In contrast, the FCR of the P5 group (1.49 ± 0.04%) significantly decreased (*p* < 0.05). The FCR of group P4 showed no significant difference compared to P1 group (*p* > 0.05) (Figure 1C).

### 3.2. The Feed Intake, Feeding Rate, and Expression of Feeding-Related Genes in Chinese Perch

**Figure 2:**
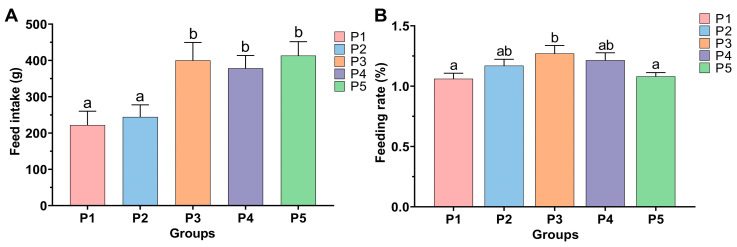
Effects of different protease supplementation levels on feed intake (**A**) and feeding rate (**B**) of Chinese perch. Different lowercase letters indicate significant differences among groups (*p* < 0.05), while identical lowercase letters indicate no significant difference (*p* > 0.05).

The effects of adding different concentrations of protease to the diet on the feeding of Chinese perch are shown in Figure 2. As shown in Figure 2A, the feed intake (FI) of the FI of P2 group (244.45 ± 33.23 g) did not differ significantly from that of the control P1 group (221.91 ± 17.85 g) (*p* > 0.05). In contrast, the FI values of P3, P4, and P5 groups were 400.25 ± 24.05 g, 378.33 ± 22.74 g, and 413.41 ± 20.49 g, respectively, all of which were significantly higher than that of P1 group (*p* < 0.05). With the increase in protease concentration, the feeding rate (FR) of each group showed a trend of first increasing and then decreasing (Figure 2B). Compared to the FR of group P1 (1.06 ± 0.05%), only the FR of P3 group (1.27 ± 0.07%) increased significantly (*p* < 0.05), while no significant difference was observed among the FR of P1, P2, P4, and P5 groups (*p* > 0.05) (Figure 2B).

**Figure 3:**
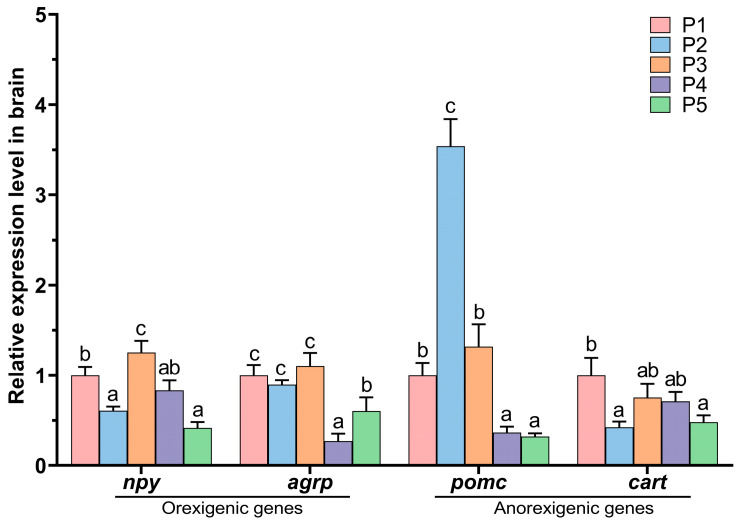
Relative expression levels of feeding-related genes in the brain in different protease-supplemented and control groups. Different lowercase letters indicate significant differences between groups (*p* < 0.05), while identical lowercase letters indicate no significant difference (*p* > 0.05).

The effect of protease concentration in feed on the expression levels of feeding-related genes in the brain of Chinese perch is illustrated in Figure 3. Compared to the control P1 group, the relative expression level of the orexigenic gene *npy* in the brains of Chinese perch in P2 and P5 groups was significantly decreased (*p* < 0.05), while the relative expression of *npy* in P3 group was significantly increased (*p* < 0.05), and there was no significant difference in the relative expression of *npy* in P4 group compared to P1 group (*p* > 0.05). Compared with the P1 group, the relative expression level of the orexigenic gene *agrp* showed no significant differences in P2 and P3 groups (*p* > 0.05), while it was significantly decreased in P4 and P5 groups (*p* < 0.05), with a more remarkable decrease in expression level in P4 group.

Compared to the P1 group, the relative expression level of the anorexigenic gene *pomc* was significantly increased in P2 group (*p* < 0.05), while it was significantly decreased in P4 and P5 groups (*p* < 0.05). Compared to the P1 group, the relative expression levels of the anorexigenic gene *cart* were significantly decreased in the P2 and P5 groups (*p* < 0.05). In contrast, although the relative expression levels of the *cart* gene were decreased in the P3 and P4 groups, there was no significant difference (*p* > 0.05) (Figure 3).

### 3.3. Protein Utilization and Deamination Gene Expression in Chinese Perch

**Figure 4:**
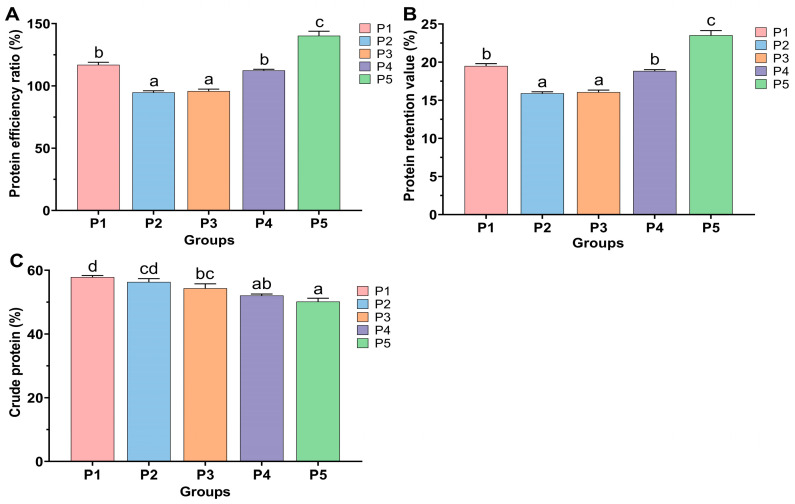
Effects of protease supplementation level on the protein efficiency ratio (**A**), protein retention value (**B**), and crude protein (**C**). Different lowercase letters indicate significant differences between groups (*p* < 0.05), while identical lowercase letters indicate no significant difference (*p* > 0.05).

The effect of protease concentration in feed on protein metabolism in Chinese perch is shown in Figure 4. Compared to the protein efficiency ratio (PER, 116.93 ± 2.05%) and protein retention value (PRV, 19.50 ± 0.30%) of the control P1 group, the protease-added groups exhibited a trend of initial decrease followed by an increase with increasing concentration. The PERs of P2 and P3 groups (94.83 ± 1.23% and 95.85 ± 1.56%, respectively) were significantly lower (*p* < 0.05) (Figure 4A), and the PRVs of P2 and P3 groups (15.90 ± 0.21% and 16.07 ± 0.26%, respectively) were also significantly lower (*p* < 0.05) (Figure 4B). Compared to the P1 group, the PER (112.41 ± 0.94) and PRV (18.85 ± 0.16) of the P4 group showed no significant difference (*p* > 0.05), while the PER (140.27 ± 3.62%) and PRV (23.53 ± 0.61%) of the P5 group were significantly increased (*p* < 0.05).

**Figure 5:**
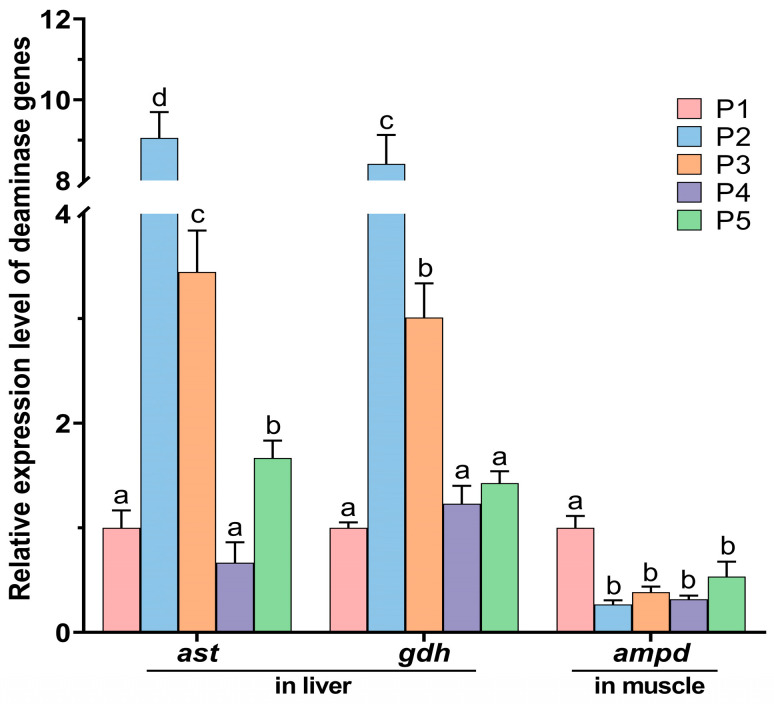
Relative expression levels of deamination-related genes in liver and muscle in different protease-supplemented and control groups. Different lowercase letters indicate significant differences between groups (*p* < 0.05), while identical lowercase letters indicate no significant difference (*p* > 0.05).

The effect of protease concentration in feed on the expression levels of deamination-related genes in the liver and muscle of Chinese perch is exhibited in Figure 5. Compared to the control P1 group, the relative expression levels of the *ast* gene in the liver of P2, P3, and P5 groups were significantly increased (*p* < 0.05), with the most significant increase observed in P2 group, while there was no significant difference in the relative expression of the *ast* gene in P4 group (*p* > 0.05). Compared to P1 group, the relative expression levels of the deamination-related gene *gdh* in the liver of P2 and P3 groups were significantly elevated (*p* < 0.05), whereas there were no significant differences in the relative expression of the *gdh* gene in P4 and P5 groups (*p* > 0.05). In the muscle of Chinese perch, the relative expression levels of the *ampd* gene in the protease supplementation groups (P2–P5) were significantly lower compared to the P1 group (*p* < 0.05).

### 3.4. Expression of Endogenous Pepsinogen Genes in Chinese Perch

**Figure 6:**
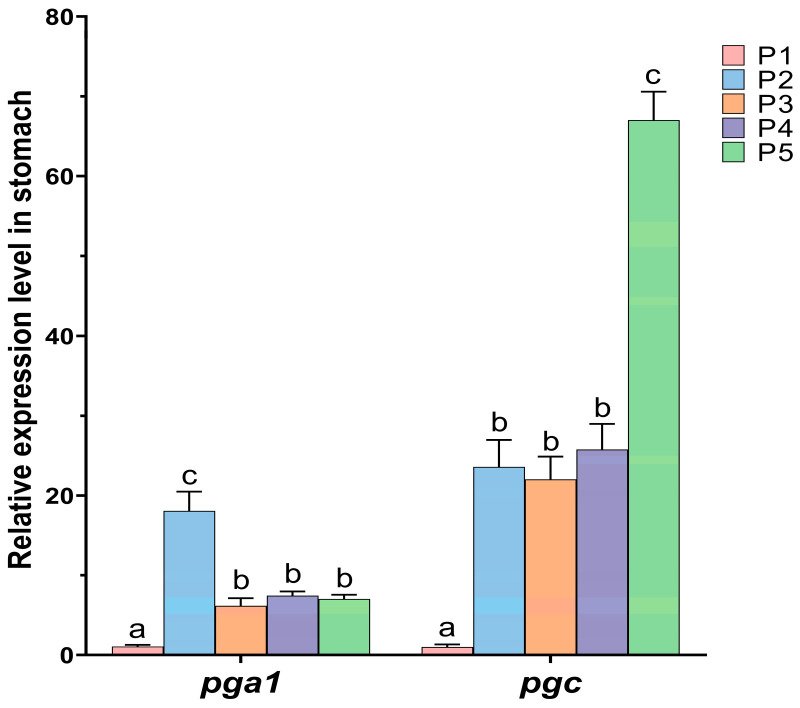
Relative expression levels of the endogenous pepsinogen genes *pga1* and *pgc* in the stomach in different protease-supplemented and control groups. Different lowercase letters indicate significant differences between groups (*p* < 0.05), while identical lowercase letters indicate no significant difference (*p* > 0.05).

The effect of protease supplementation in feed on the expression of endogenous pepsinogen genes in the stomach of Chinese perch is shown in Figure 6.

Compared with the control P1 group, the relative expression levels of the endogenous pepsinogen *pga1* gene in the protease-supplemented groups (P2–P5) were significantly increased (*p* < 0.05), and the expression level of the *pga1* gene in P2 group was significantly higher than that in the other four groups (*p* < 0.05). Compared with the control P1 group, the relative expression levels of the endogenous pepsinogen *pgc* gene in the protease-supplemented groups (P2–P5) were significantly increased (*p* < 0.05), and the expression level of the *pgc* gene in P5 group was significantly higher than that in the other four groups (*p* < 0.05).

### 3.5. Expression of Glucose and Lipid Metabolism Genes in Chinese Perch

**Figure 7:**
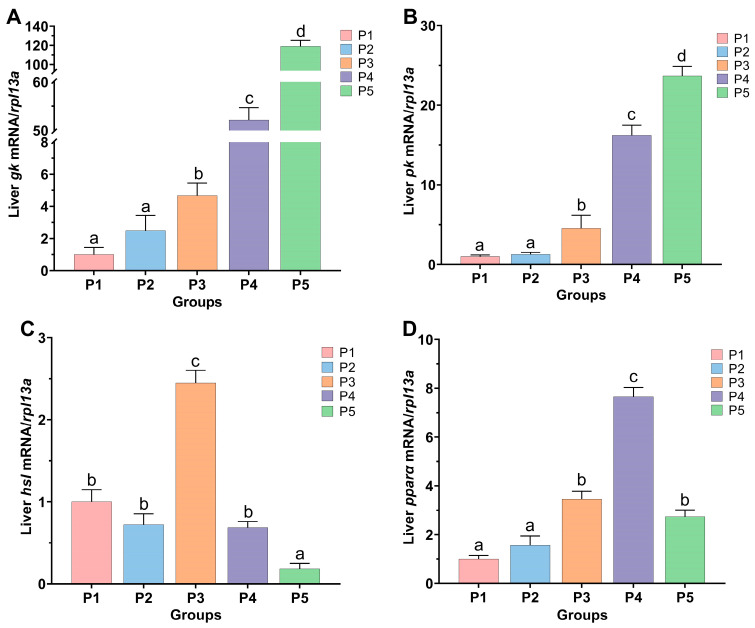
Relative expression levels of glycolipid-related genes *gk* (**A**), *pk* (**B**), *hsl* (**C**), and *pparα* (**D**) in the liver in different protease-supplemented and control groups. Different lowercase letters indicate significant differences between groups (*p* < 0.05), while identical lowercase letters indicate no significant difference (*p* > 0.05).

The effect of protease supplementation in feed on the expression levels of genes related to glucose and lipid metabolism in the liver of Chinese perch is shown in Figure 7. As the dietary protease concentration increased, the expression levels of the glycolytic genes *gk* and *pk* also showed a gradual increasing trend (Figure 7A,B). Compared to the control P1 group, the expression levels of *gk* and *pk* in the liver were significantly increased in P3, P4, and P5 groups (*p* < 0.05). Furthermore, the relative expression levels of *gk* and *pk* in P5 group were significantly higher than those in P3 and P4 groups (*p* < 0.05). In contrast, the relative expression levels of *gk* and *pk* in P2 group showed no significant differences compared to P1 group (*p* > 0.05).

As shown in Figure 7C,D, compared with the control P1 group, the relative expression level of the lipolysis-related gene *hsl* in the liver of P3 group was significantly increased (*p* < 0.05), while the relative expression level of the *hsl* gene in P5 group was significantly decreased (*p* < 0.05). There was no significant difference in the relative expression level of the *hsl* gene between P2 and P4 groups (*p* > 0.05). Compared with P1 group, the relative expression levels of the lipolysis-related gene *ppar*α in the liver of P3, P4, and P5 groups were all significantly increased (*p* < 0.05), while there was no significant difference in the expression level of the *pparα* gene in P2 group (*p* > 0.05).

## 4. Discussion

### 4.1. Effects of Dietary Protease Levels on Feed Utilization and Growth in Chinese Perch

**Figure 8:**
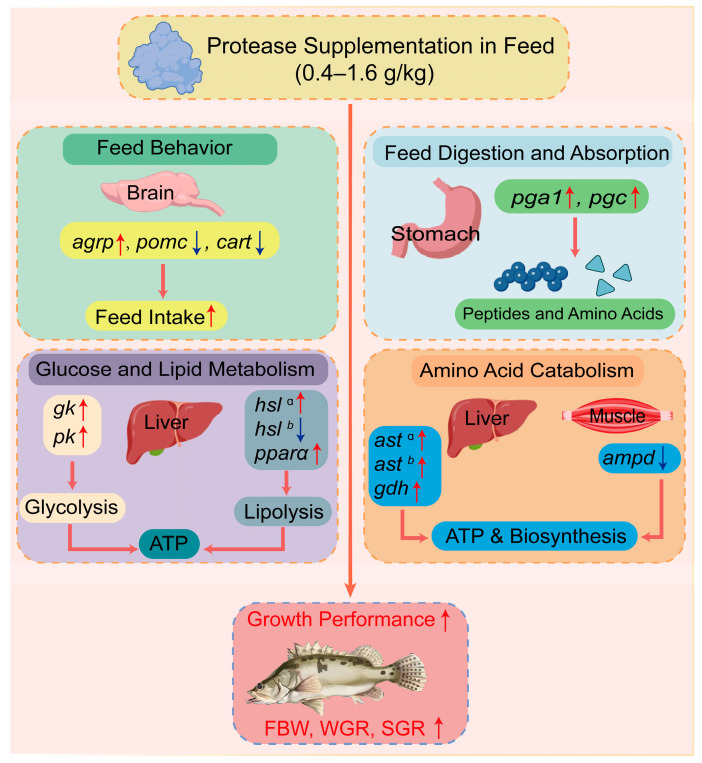
The mechanism of protease supplementation in feed to enhance the growth performance of Chinese perch. The superscript lowercase letter “a” indicates a protease addition level of 0.4 g/kg; the superscript lowercase letter “b” indicates a protease addition level of 0.8 g/kg. Red arrows indicate an increase in the parameters or expression levels of related genes in the pathway. Blue arrows represent a decrease in the expression levels of related genes in the pathway.

The results of this study indicated that dietary supplementation with low concentrations of protease (0.2–0.4 g/kg) resulted in no significant differences in weight gain rate (WGR) and specific growth rate (SGR) of Chinese perch. However, when supplemented with a high concentration of protease (1.6 g/kg), the WGR and SGR of Chinese perch were significantly higher than those in the control group and the low-concentration protease (0.2–0.4 g/kg) supplementation group. Additionally, the feed conversion ratio (FCR) was significantly lower than that of the control group (*p* < 0.05), indicating that dietary supplementation with higher concentrations of protease can significantly improve the growth performance of Chinese perch (Figure 8). Similar results have been reported in studies by Shi et al. and Adeoye et al., where dietary supplementation with 0.15–0.175 g/kg protease in gibel carp (*C. gibelio*) [25] and 0.2 g/kg protease in Nile tilapia (*O. niloticus*) [47] increased both WGR and SGR. Furthermore, only at the protease supplementation concentration of 1.6 g/kg, the protein efficiency ratio (PER) and protein retention value (PRV) of Chinese perch were significantly higher than those in the control group (*p* < 0.05), while the crude protein (CP) content was significantly lower than that in the control group (*p* < 0.05). Similar findings have also been reported in studies on Nile tilapia (*O. niloticus*), where dietary supplementation with 1.0 and 1.5 g/kg of complex enzymes (neutral protease, β-glucanase, and xylanase) in a 30.1% protein diet significantly increased the SGR, feed efficiency ratio, and apparent digestibility coefficient of protein in Nile tilapia (*O. niloticus*) [19].

However, the addition of low-concentration protease (0.2–0.4 g/kg) significantly increased the FCR of Chinese perch, but decreased its PER and PRV (*p* < 0.05). This phenomenon may be attributed to the significant up-regulation of hepatic aminotransferase genes (*ast* and *gdh*) induced by the low-concentration protease (Figure 5), as increased expression of deamination genes in the liver may enhance amino acid catabolism [48]. Although the increased feed intake in the group supplemented with 0.4 g/kg protease somewhat elevated the amino acid levels of the fish, the overall amino acid catabolism exceeded protein biosynthesis. This consequently exerted a negative regulatory effect on growth performance, ultimately manifesting as decreased PER and PRV. In contrast, studies supplementing different concentrations (0.075–0.75 g/kg) of protease to low-protein diets (30.4 g/kg) for rohu (*Labeo rohita*) and high-protein diets (338 g/kg) for gibel carp (*C. gibelio*) juveniles found that protease concentrations around 0.4 g/kg improved growth performance, crude protein content, and feed utilization efficiency in both species, with no adverse effects on fish health [11,49].

In summary, the optimal protease supplementation level in feed is closely related to fish species, developmental stage, and dietary protein content. In this study, the improvement in the growth performance of Chinese perch with 1.6 g/kg protease supplementation may be attributed to its ability to facilitate the hydrolysis of macromolecular proteins into small-molecule peptides and various amino acids for digestion and absorption by the fish [10,25]. This process reduces gastrointestinal irritation and nutrient absorption impairment caused by the feed, enhances feed utilization efficiency, and consequently promotes fish growth. However, supplementing feed with low concentrations (0.2–0.4 g/kg) of protease has been shown to exert negative effects on the growth performance of Chinese perch [20,50].

### 4.2. Effects of Protease Supplementation Levels in Feed on the Feed Intake of Chinese Perch

The growth performance of fish is directly related to feed intake, and the feeding behavior of fish is primarily regulated by various feeding factors [51], mainly including orexigenic factors (agouti gene-related protein, AgRP and neuropeptide Y, NPY) and anorexigenic factors (proopiomelanocortin, PMOC and cocaine amphetamine-regulated transcript, CART). They can act as signaling factors to regulate multiple signaling pathways, thereby collectively modulating fish appetite. This balancing mechanism is crucial for maintaining energy homeostasis in fish [51,52]. In this study, dietary supplementation with 0.4–1.6 g/kg protease significantly increased the feed intake (FI) of Chinese perch (*p* < 0.05), while the feeding rate (FR) significantly increased only at 0.4 g/kg supplementation (*p* < 0.05), followed by a gradual decreasing trend. qRT-PCR results indicated that 0.4 g/kg dietary protease up-regulated the expression of orexigenic genes *npy* and *agrp*, while 0.8–1.6 g/kg protease down-regulated the expression of anorexigenic genes *pomc* and *cart*, thereby promoting feed intake in Chinese perch (Figure 3 and Figure 8).

However, when 0.2 g/kg of protease was added to the feed, although the feed intake of Chinese perch showed no significant change, the expression level of the orexigenic gene *npy* significantly decreased (*p* < 0.05), while the expression level of the anorexigenic gene *pomc* significantly increased (*p* < 0.05). These results indicate that the addition of low-concentration exogenous protease may have a negative impact on the feeding behavior of Chinese perch, consequently affecting the growth performance of the fish. Similar results have been reported in studies on Nile tilapia (*O. niloticus*), where the addition of 0.5 g/kg of a compound enzyme (protease, pepsin, trypsin, xylanase, and amylase) resulted in a certain decrease in feed intake compared to the control group, but an increase in growth performance [53].

In conclusion, the addition of low-concentration protease (0.4 g/kg) to the feed in this study promoted feeding in Chinese perch by enhancing the expression of orexigenic genes, while medium-to-high concentrations (0.8–1.6 g/kg) promoted feeding by suppressing the expression of anorexigenic genes. Conversely, the addition of low-concentration protease (0.2 g/kg) not only inhibited the expression of orexigenic genes but also promoted the expression of orexigenic genes, thereby exerting negative effects on Chinese perch feeding and ultimately impacting growth performance.

### 4.3. Effects of Dietary Protease Supplementation Level on the Expression of Transamination and Deamination Genes in Chinese Perch

As a typical carnivorous fish, Chinese perch requires substantial protein intake. Protein intake is positively correlated with ammonia excretion, and ammonia accounts for no less than 80% of the total nitrogen excretion [38,54]. Due to the high sensitivity of Chinese perch to ammonia nitrogen [37] its ammonia metabolism occurs primarily in the liver, mediated mainly by various transaminases and deaminases [55]. In this study, supplementation with low concentrations (0.2–0.4 g/kg) of protease significantly increased the expression levels of the glutamate dehydrogenase (GDH) gene (*gdh*) and aspartate aminotransferase (AST) gene (*ast*) in the liver (*p* < 0.05). This result is likely attributable to the protease supplementation promoting feeding and the secretion of endogenous gastric protease in Chinese perch (Figure 2A and Figure 6), thereby accelerating protein breakdown into peptides, enhancing protein digestion and absorption, and consequently elevating amino acid levels within the fish [45]. In the liver, dietary amino acids exceeding the requirements for growth and protein synthesis are degraded preferentially to carbohydrates and lipids [13]. Therefore, amino acid metabolism is accelerated in the liver by promoting the expression of deamination genes. Karlsson et al. and Tng et al. found that the accumulation of amino acids increased in the intestines and liver of rainbow trout (*Oncorhynchus mykiss*) [56] and juvenile marble goby (*Oxyeleotris marmorata*) [57] after feeding, and the activity of glutamate dehydrogenase (GDH) in the intestines and livers of marble goby (*O. marmorata*) significantly increased. Furthermore, Liu et al. found that supplementing feed with a relatively low concentration (0.075 g/kg) of neutral protease resulted in the highest AST activity in the hepatopancreas of juvenile gibel carp (*C. gibelio*) [11], which aligns with our findings.

However, compared with the addition of 0.2 g/kg, when the dietary protease supplementation level was 0.4–1.6 g/kg, the relative expression levels of the *ast* and *gdh* genes significantly decreased (*p* < 0.05). This indicated that adding 0.4–1.6 g/kg of protease significantly reduced the transamination and deamination capacity in Chinese perch, thereby weakening the promotion of amino acid catabolism and leading to reduced energy supply from amino acid catabolism. When 1.6 g/kg of protease was added to the diet, the WGR, SGR, PER, PRV, and the expression levels of glucose-lipid metabolism genes all significantly increased, while the FCR significantly decreased (*p* < 0.05). This suggested that while the amino acid catabolic pathway was weakened, the protein biosynthesis pathway was enhanced (Figure 8). Therefore, adding a high concentration of protease (1.6 g/kg) to Chinese perch aquaculture feed can improve the utilization efficiency of protein and amino acids, enhance protein synthesis capacity, and consequently promote the growth of Chinese perch.

Under aerobic conditions, ammonia metabolism in muscle depends on the fish’s activity level and movement intensity, whereas under normal conditions, most ammonia is produced in the liver [58]. Under conditions of energy insufficiency, muscle amino acids are degraded. This degradation triggers elevated expression of deaminase genes within the muscle tissue [13]. In this study, supplementation with different concentrations of protease significantly inhibited the expression level of the adenosine monophosphate deaminase (AMPD) gene (*ampd*) in muscle (*p* < 0.05). It is speculated that increased feed intake may facilitate greater protein breakdown into amino acids, thereby promoting hepatic catabolism of amino acids to supply energy. Consequently, the breakdown of muscle-derived amino acids becomes unnecessary [59]. Furthermore, protease supplementation significantly reduced the expression of deamination-related genes in the muscle of Chinese perch, effectively preventing elevated blood ammonia levels and subsequent ammonia toxicity [60].

### 4.4. Effects of Protease Supplementation Level in Feed on Endogenous Pepsin Secretion in Chinese Perch

Pepsin is one of the most important digestive enzymes in fish, serving as a crucial indicator of gastric digestive capacity in carnivorous fish. It plays a pivotal role in the digestion and absorption of protein, thereby influencing fish growth and development [61]. Pepsinogens in fish are primarily classified into two categories Pepsinogen A (PGA) and Pepsinogen C (PGC), which can be converted into active pepsin under acidic conditions [62]. Therefore, proteases need to be supplemented in feed to compensate for the deficiency of endogenous proteases. The results of this study showed that the relative expression levels of the pepsinogen genes *pga1* and *pgc* in the protease-supplemented groups (0.2–1.6 g/kg) were both significantly increased compared to the control group (*p* < 0.05). Similar results have also been reported in studies on snakehead (*C. argus*) and Nile tilapia (*O. niloticus*), where protease supplementation promoted the secretion of endogenous digestive enzymes (pepsin, amylase, chymotrypsin, and trypsin) and enhanced enzyme activity [3,12,28,53].

Among all protease supplementation groups, the addition of 0.2 g/kg protease to the feed most significantly increased the expression level of the *pga1* gene (*p* < 0.05). However, its enhancement of *pgc* gene expression was less significant than the 1.6 g/kg protease supplementation group. Furthermore, high-concentration protease can directly stimulate feeding behavior and activate the activation and secretion of pepsinogen, accelerating the protein decomposition process (Figure 8). Monier also reported in common carp (*C. carpio*) feed that adding 1.0 g/kg of an exogenous enzyme mixture (Hostazyme X) significantly increased the activity of amylase, lipase, and protease in the common carp intestine, consequently increasing the FI, WGR, and SGR of common carp [16]. Furthermore, Ding et al. reported that supplementing the diet of snakehead (*C. argus*) with an appropriate amount of a compound enzyme complex (800 U/kg amylase, 300 U/kg acid protease, and 3800 U/kg neutral protease) significantly enhanced the expression of the pepsinogen gene. This was accompanied by increased activities of pepsin and other digestive enzymes, ultimately leading to improved growth performance in snakehead (*C. argus*) [63]. Similarly, Islam et al. observed that the addition of 0.5 g/kg gastric protease to the diet of striped catfish (*Pangasianodon hypophthalmus*) resulted in significantly enhanced growth performance and a remarkable increase in the PER [64]. In this study, WGR, SGR, and FI of Chinese perch also increased significantly when 1.6 g/kg protease was supplemented. This indicated that supplementing 1.6 g/kg protease stimulated the expression of endogenous protease genes, potentially enhancing nutrient digestibility by increasing protease activity in the gastrointestinal tract, thereby further promoting the growth performance of Chinese perch.

### 4.5. Effects of Protease Supplementation in Feed on Glucose and Lipid Metabolism in Chinese Perch

Carbohydrates and lipids are important energy-supplying substances in fish [38,40]. Under aerobic conditions, glucose is completely catabolized through glycolysis, the tricarboxylic acid cycle, and the electron transport chain to produce ATP, or it enters the pentose phosphate pathway leading to the production of nicotinamide adenine dinucleotide phosphate for biosynthetic purposes [65]. Excess glucose can be stored as glycogen or converted into lipids. When the catabolism of carbohydrates and lipids is enhanced, the energy supply in fish is sufficient, which is beneficial for protein synthesis and deposition, thereby promoting fish growth [38,40]. Glucokinase (GK), pyruvate kinase (PK), and phosphoenolpyruvate carboxykinase are key enzymes involved in the glycolysis and gluconeogenesis pathways [66,67]. In this study, dietary supplementation with protease at 0.4–1.6 g/kg significantly increased the relative expression levels of hepatic glycolysis genes (*gk* and *pk*) (*p* < 0.05), and the relative expression levels of *gk* and *pk* genes increased with increasing enzyme concentration, indicating that protease supplementation promoted carbohydrate catabolism in Chinese perch. Similar results have been reported in studies on largemouth bass (*Micropterus salmoides*) and snakehead (*C. argus*): dietary supplementation with protease (4500 U/kg) significantly increased the expression levels of *gk* and *pepck* genes in the liver of largemouth bass (*M. salmoides*) [68]; supplementation with a complex enzyme (6700 U/kg amylase, 8000 U/kg acid protease, and 50,000 U/kg neutral protease) in the diet of snakehead (*C. argus*) enhanced the expression levels of its carbohydrate metabolism-related genes [63].

Hormone-sensitive lipase (HSL) hydrolyzes lipids stored in fish adipose tissue into free fatty acids, releasing them into the bloodstream to provide an energy source for most tissues [69]. Peroxisome proliferator-activated receptor α (PPARα), belonging to the PPAR nuclear receptor family, regulates the gene expression of multiple lipid catabolic pathways [70,71]. In this study, the expression level of the *hsl* gene significantly increased at a dietary protease concentration of 0.4 g/kg but significantly decreased at 1.6 g/kg (*p* < 0.05). At protease concentrations ranging from 0.4 to 1.6 g/kg, the expression level of the *pparα* gene initially increased and subsequently decreased; however, its expression level remained significantly higher than that of the control group (*p* < 0.05). The results of Guan et al. showed that adding 4500 U/kg of protease to largemouth bass (*M. salmoides*) significantly increased the gene expression levels of *hsl* and *pparα*, while adding 7500 U/kg of protease resulted in decreased *pparα* gene expression, which is highly consistent with our findings [68]. This phenomenon may occur because at lower dietary protease concentrations, the capacity for carbohydrate catabolism is weaker, leading to insufficient energy supply for the fish body, thereby up-regulating *hsl* gene expression in the liver [72]. When higher concentrations of protease are added to the feed, Chinese perch do not need to secrete HSL to break down existing lipids; instead, they convert amino acids into glycogen and lipids for storage. Therefore, adding an appropriate concentration of protease can not only promote feeding in Chinese perch and enhance glycolysis efficiency by up-regulating the expression levels of key genes involved in carbohydrate metabolism (Figure 8), but also prevent lipid accumulation by regulating the expression levels of genes related to lipid catabolism. Ultimately, this synergistically promotes the growth performance of Chinese perch.

### 4.6. Outlook

This study systematically evaluated the effects of dietary protease levels on the growth performance, expression of appetite-regulating genes, pepsinogen genes, deamination-related genes, and key glycolipid metabolism genes in Chinese perch. The results showed that the addition of 1.6 g/kg protease significantly enhanced the growth performance and feed utilization efficiency of Chinese perch. However, establishing additional experimental groups with concentrations higher than 1.6 g/kg is necessary to determine whether this concentration represents the optimum. Besides stomach, the intestine also plays a crucial role in the digestion and absorption of feed. Therefore, future research will aim to determine the optimal concentration of protease and systematically elucidate the effects of dietary protease on the intestinal morphology, antioxidant capacity, intercellular tight junction-related and pro-inflammatory cytokine-related gene expression.

## 5. Conclusions

This study found that dietary supplementation with 1.6 g/kg protease significantly improved the growth performance (WGR, SGR, PER, and PRV) of Chinese perch and significantly reduced the FCR. However, supplementation with 0.2 g/kg protease inhibited the growth performance. Supplementation with 1.6 g/kg protease up-regulated the expression of pepsinogen genes (*pga1* and *pgc*), hepatic deamination genes (*ast*), carbohydrate metabolism genes (*gk* and *pk*), and lipid metabolism genes (*pparα*), while down-regulating the expression of anorexigenic genes (*pomc* and *cart*), muscular deamination gene (*ampd*), and lipid metabolism gene (*hsl*). Therefore, supplementing with 1.6 g/kg protease improves feed utilization, enhances feeding and protein digestion and absorption in Chinese perch, accelerates the catabolism of carbohydrates and lipids, increases energy supply while reducing hepatic lipid accumulation, enhances hepatic amino acid utilization while inhibiting muscular protein degradation, promotes protein synthesis and deposition, and ultimately improves the growth performance of Chinese perch.

## Figures and Tables

**Table 1:** Nutritional Composition of Chinese perch Feed.

Ingredient Composition (g/kg)	P1	P2	P3	P4	P5
Fishmeal	400	400	400	400	400
Corn gluten meal	300	300	300	300	300
Fish oil	50	50	50	50	50
Corn starch	50	50	50	50	50
Amino acid premix ^1^	50	50	50	50	50
Mineral premix ^2^	20	20	20	20	20
Vitamin premix ^3^	20	20	20	20	20
Monocalcium phosphate	20	20	20	20	20
Carboxymethyl cellulose	30	30	30	30	30
Choline chloride (50%)	5	5	5	5	5
Microcrystalline cellulose	55	54.8	54.6	54.2	53.4
Protease ^4^	0	0.2	0.4	0.8	1.6
Total	1000	1000	1000	1000	1000
Nutritional composition					
Crude protein	478.8	478.8	478.8	478.8	478.8
Lysine (Lys)	21.03	21.03	21.03	21.03	21.03
Methionine (Met)	11.46	11.46	11.46	11.46	11.46
Crude lipid	85	85	85	85	85
Carbohydrate	95	95	95	95	95

^1^ Amino acid mix (per kg of diet): Thr 9.0 g; Arg 9.5 g; Trp 3.5 g; Ile 7.0 g; Leu 0 g; Val 10 g; His 3.5 g; Phe 3.0 g; Cys 2.5 g; Tyr 2.0 g. ^2^ Mineral premix (per kg of diet): CaHPO_4_ 94.9 g, KCl 5.45 g, MgSO_4_ 4 g, NaCl 3.8 mg, CuSO_4_ 25 mg, FeSO_4_ 407 mg, ZnSO_4_ 198 mg, MnSO_4_ 36 mg, Na_2_SeO_3_ 1.8 mg, KI 1.4 mg, Na_2_MoO_4_ 0.34 mg, CoSO_4_ 0.09 mg, KF 0.8 mg. ^3^ Vitamin premix (per kg of diet): inositol 600 mg, vitamin A 40 mg, vitamin D_3_ 0.06 mg, vitamin E 200 mg, vitamin K_3_ 10 mg, vitamin B_1_ (thiamine) 15 mg, vitamin B_2_ (riboflavin) 25 mg, vitamin B_6_ 20 mg, pantothenic acid 50 mg, vitamin B_3_ (nicotinic acid) 200 mg, biotin 3.2 mg, vitamin B_12_ 0.1 mg, folic acid 10 mg, vitamin C 210 mg. ^4^ Experimental protease (EC3.4.23.18) (purchased from Wuhan SunHY Biology Co., Ltd.).

**Table 2:** qRT-PCR primer sequence for Chinese perch.

Gene	Primer	Primer Sequence (5′–3′)	E-Value (%)	Tm (°C)	Product Size (bp)	Accession Number
*gdh*	F R	GACGACGACCCCAACTTCT GACCCGCTTCCTCTTCTGC	94.5	58	126	XM_044213922.1
*ast*	F R	TGGGTATTATGTGCTGGTCA CTTCTTGGTAAAGTGCCTCA	98.9	58	131	XM_044190330
*ampd*	F R	CATTTCCTTCCCGTGTT TCTGTCTGCGGAGTTGGT	103.6	58	242	XM_044212879
*gk*	F R	AAGGTGGAGACCAAGAAC TGCCCTTGTCAATGTCC	96.9	58	190	MW140068.1
*pk*	F R	CGCCCTCGCTGTCCTATTA TGCCGAAGTTGACCCTGTTG	99.9	57	173	XM_044207998.1
*pga1*	F R	CCAGAACGGAGACTATGT GTATTGAGACTGACGGAC	104	59	267	EU807930.1
*pgc*	F R	CTACGCTGATACCACCTA GTTACAGTAGACGGAGTC	96.4	59	130	EU807929.1
*agrp*	F R	GTGCTGCTCTGCTGTTGG AGGTGTCACAGGGGTCGC	104	65	295	XM_044195210.1
*npy*	F R	GGAAGGATACCCGGTGAAA TCTTGACTGTGGAATCGTG	107.2	52	202	XM_044172804.1
*pomc*	F R	GGCTGAAGATGGTGTGTCTATG ACATGCAGAGGTGAATACAGTC	97.7	58	268	XM_044187638.1
*cart*	F R	TCTGCACGAAGTGTTGGA GCACATCTTCCCGATACGA	105	56	171	XM_044192257.1
*hsl*	F R	ACAAACGCCTGGGAATGGT TGTGGTCCGCCCTGAAGAA	99.6	58	125	XM_044208783.1
*pparα*	F R	GGGTGTGCTCAGACAAGGCT GTTGCGGTTCTTCTTTTGGAT	105.4	58	146	XM_044194385.1
*rpl13a*	F R	CACCCTATGACAAGAGGAAGC TGTGCCAGACGCCCAAG	102.9	59	100	XM_044166826.1

Note: Tm: melting temperature.

## Data Availability

The original contributions presented in this study are included in the article. Further inquiries can be directed to the corresponding authors.

## Abbreviations

The following abbreviations are used in this manuscript:

AgRP	Agouti gene-related protein
AMPD	Adenosine monophosphate deaminase
AST	Aspartate aminotransferase
CART	Cocaine amphetamine-regulated transcript
CP	Crude protein
FCR	Feed conversion rate
FI	Feed intake
FR	Feed rate
GDH	Glutamate dehydrogenase
GK	Glucokinase
HSL	Hormone-sensitive lipase
NPY	Neuropeptide Y
PER	Protein efficiency ratio
PGA	Pepsinogen A
PGC	Pepsinogen C
PK	Pyruvate kinase
PMOC	Proopiomelanocortin
PPARα	Peroxisome proliferator-activated receptor α
PRV	Protein retention value
SGR	Specific growth rate
WGR	Weight gain rate
